# Preconditioning-Induced Facilitation of Lactate Release from Astrocytes Is Essential for Brain Ischemic Tolerance

**DOI:** 10.1523/ENEURO.0494-23.2024

**Published:** 2024-04-23

**Authors:** Yuri Hirayama, Ha Pham Ngoc Le, Hirofumi Hashimoto, Itsuko Ishii, Schuichi Koizumi, Naohiko Anzai

**Affiliations:** ^1^Department of Pharmacology, Chiba University Graduate School of Medicine, Chiba 260-8670, Japan; ^2^Department of Neuropharmacology, Interdisciplinary Graduate School of Medicine, University of Yamanashi, Yamanashi 409-3898, Japan; ^3^Division of Pharmacy, Chiba University Hospital, Chiba 260-8677, Japan; ^4^GLIA Center, University of Yamanashi, Yamanashi 409-3898, Japan

**Keywords:** astrocytes, CD147, ischemic tolerance, lactate, P2X7 receptor

## Abstract

A sublethal ischemic episode [termed preconditioning (PC)] protects neurons in the brain against a subsequent severe ischemic injury. This phenomenon is known as brain ischemic tolerance and has received much attention from researchers because of its robust neuroprotective effects. We have previously reported that PC activates astrocytes and subsequently upregulates P2X7 receptors, thereby leading to ischemic tolerance. However, the downstream signals of P2X7 receptors that are responsible for PC-induced ischemic tolerance remain unknown. Here, we show that PC-induced P2X7 receptor-mediated lactate release from astrocytes has an indispensable role in this event. Using a transient focal cerebral ischemia model caused by middle cerebral artery occlusion, extracellular lactate levels during severe ischemia were significantly increased in mice who experienced PC; this increase was dependent on P2X7 receptors. In addition, the intracerebroventricular injection of lactate protected against cerebral ischemic injury. In in vitro experiments, although stimulation of astrocytes with the P2X7 receptor agonist BzATP had no effect on the protein levels of monocarboxylate transporter (MCT) 1 and MCT4 (which are responsible for lactate release from astrocytes), BzATP induced the plasma membrane translocation of these MCTs via their chaperone CD147. Importantly, CD147 was increased in activated astrocytes after PC, and CD147-blocking antibody abolished the PC-induced facilitation of astrocytic lactate release and ischemic tolerance. Taken together, our findings suggest that astrocytes induce ischemic tolerance via P2X7 receptor-mediated lactate release.

## Significance Statement

Brain ischemic tolerance refers to an endogenous neuroprotective phenomenon whereby a nonlethal ischemic episode, termed preconditioning (PC), induces resistance to a subsequent severe ischemic injury. This phenomenon has received much attention because of its robust neuroprotective effects. We have previously reported that the PC-evoked activation of astrocytes leads to ischemic tolerance; however, the underlying molecular mechanisms remain unknown. Here, we have demonstrated that PC induces the membrane translocation of lactate transporters in activated astrocytes, thereby promoting lactate release from astrocytes during severe ischemia; this effect likely plays a role in ischemic tolerance. These findings may facilitate the development of new therapeutic strategies for cerebral ischemia.

## Introduction

Lactate is an end product of glycolysis and glycogenolysis and has been considered a toxic waste product because intracellular lactate accumulation leads to pH reduction and acidosis. It has recently been reported that lactate released into the extracellular space plays a key role as a modulator of multiple cellular processes via its transporters [monocarboxylate transporters (MCTs)] and receptor [hydroxycarboxylic acid receptor 1 (HCA1); Sun et al., 2017]. In cerebral ischemia, abundant lactate is produced by astrocytes, which store glycogen (a major source of lactate in ischemia); the astrocytes release the lactate into the extracellular space via MCT1 and MCT4 (Zhang et al., 2022). Although several reports indicate that extracellular lactate is neuroprotective in cerebral ischemic injury (Berthet et al., 2009; Horn and Klein, 2013), the role of lactate in brain ischemic tolerance remains unknown.

Brain ischemic tolerance refers to an endogenous neuroprotective phenomenon whereby an experience of nonlethal ischemic episode [termed preconditioning (PC)] induces resistance to a subsequent severe ischemic injury. This phenomenon has received much attention because of its robust neuroprotective effects (Gidday, 2006; Dirnagl et al., 2009). We have reported that PC-evoked astrocytic activation induces ischemic tolerance via the P2X7 receptor/hypoxia-inducible factor-1α (HIF-1α) signaling pathway (Hirayama et al., 2015, 2021; Hirayama and Koizumi, 2017). The transcription factor HIF-1α, referred to as a “master regulator of oxygen homeostasis,” promotes the expression of hundreds of genes involved in angiogenesis and numerous other processes in a cell type-specific manner (Baranova et al., 2007; Taylor and Scholz, 2022). HIF-1α also regulates the expression of genes that are involved in lactate production and release (Semenza, 2013). It is therefore possible that the PC-induced facilitation of lactate release from astrocytes via the P2X7 receptor/HIF-1α signaling pathway can induce neuroprotection, thereby leading to ischemic tolerance. However, there is little information regarding the involvement of astrocytic lactate release in PC-induced ischemic tolerance.

In the present study, we revealed that the P2X7 receptor/HIF-1α signaling pathway in astrocytes induces the membrane translocation of MCT1 and MCT4 via CD147, which is a chaperone of these MCTs. Our findings indicate that a PC-induced increase in CD147 in astrocytes may facilitate astrocytic lactate release during severe ischemia, thus inducing ischemic tolerance.

## Materials and Methods

### Animals

P2X7 receptor knock-out mice (Solle et al., 2001; P2X7^−/−^ mice, on a C57BL/6 background) were kindly provided by Drs. Hiroshi Enaida (Saga University, Saga, Japan) and Shoji Notomi (Kyushu University, Fukuoka, Japan). C57BL/6J mice (8–10 weeks old) were purchased from Japan SLC. Animals were allowed *ad libitum* access to food and water and were maintained under temperature-, humidity-, and light-controlled conditions. Male mice were used for all experiments. The procedures using P2X7^−/−^ mice were approved by the Animal Care Committee of Yamanashi University (Yamanashi, Japan; Approval No: A23-9) and performed in accordance with the “Guiding Principles for the Care and Use of Animals in the Field of Physiologic Sciences,” published by the Physiological Society of Japan. All other procedures were approved by the Chiba University Institutional Animal Care and Use Committee (Chiba, Japan; Approval No: A5-005 and A5-006).

### Brain ischemic tolerance model

Unilateral transient focal ischemia was induced in mice by right middle cerebral artery occlusion (MCAO) with an intraluminal filament, as previously described (Ikeda-Matsuo et al., 2006; Morizawa et al., 2017). The mice were anesthetized with 4% [volume/volume (v/v)] isoflurane and maintained at 2% (v/v) isoflurane with a facemask. A neck incision was made, and the common carotid artery, internal carotid artery, and external carotid artery were exposed by dissection. Subsequently, the external carotid artery was ligated, and an 11 mm length of 6-0 silicone-coated monofilament suture was inserted into the internal carotid artery via the proximal external carotid artery, and then into the circle of Willis, thereby occluding the MCA. For PC, the MCA was occluded for 15 min; the suture was then carefully withdrawn to allow reperfusion of the ischemic region. Three days later, the same suture was inserted to occlude the MCA again, this time for 1 h (to induce severe MCAO). The suture was then carefully withdrawn, and the mice were allowed to survive for 3 d. Sham-operated mice were subjected to similar surgical procedures but without MCAO. Body temperature was monitored by a rectal thermometer and maintained at 37°C using a warm pad during surgery. Prior to reperfusion, the animals were scored for neurological deficits as follows: 0, no deficit; 1, flexion of the torso and contralateral forelimb for <3 s when lifted by the tail; 2, circling to the affected side while walking; 3, flexion of the torso and contralateral forelimb for >3 s when lifted by the tail; 4, contralateral forelimb weakness upon application of pressure to the side of the body; 5, circling to the affected side using only the forelimb; and 6, no spontaneous locomotor activity. Animals were excluded from the data collection and analysis if their behavioral scores were <4.

### Drug administration

**Figure 1.**
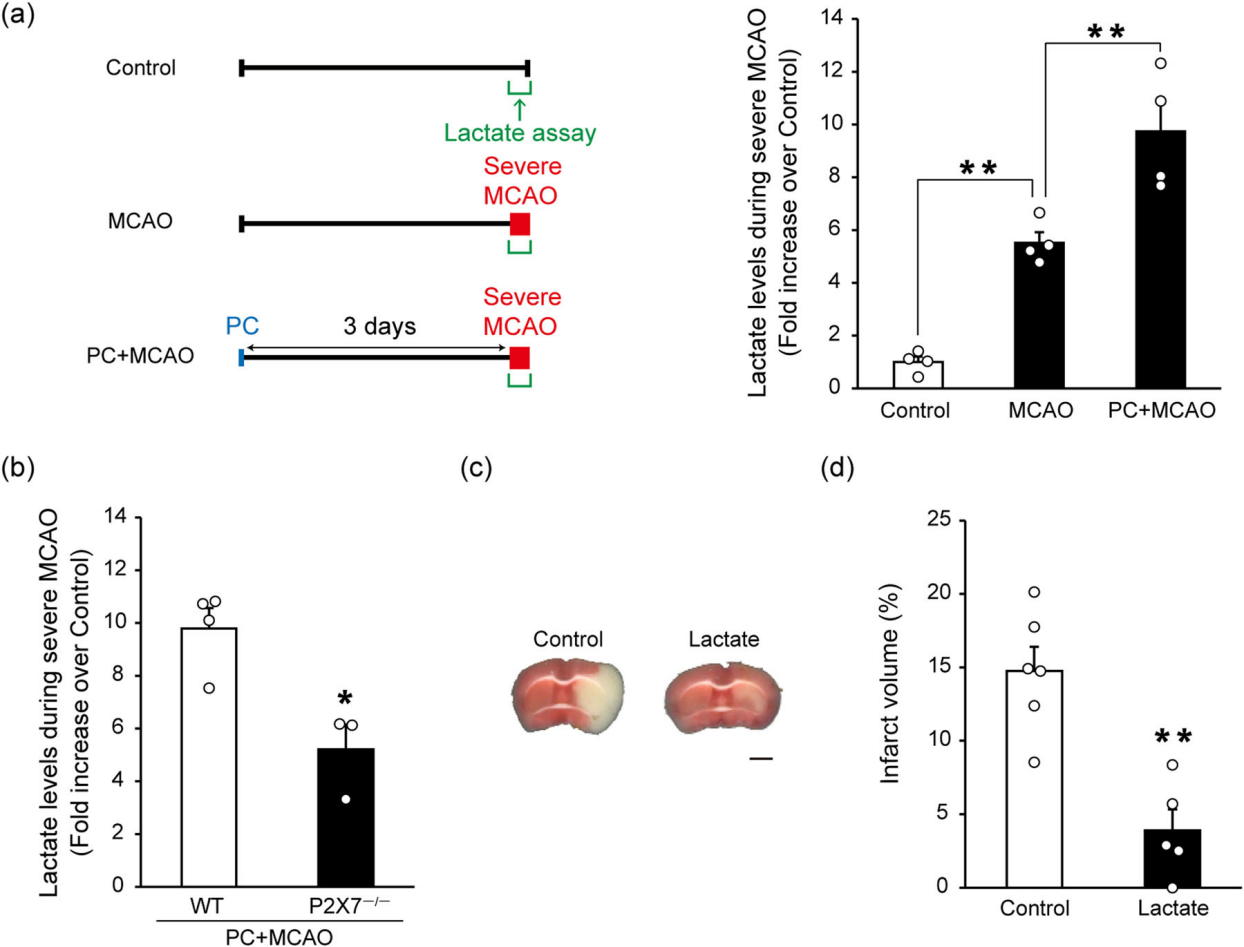
P2X7 receptor-mediated increase in extracellular lactate levels during severe ischemia and the neuroprotective effects of lactate on cerebral ischemic injury. ***a***, ***b***, In the in vivo microdialysis experiment, a probe was inserted into the ipsilateral striatum. Artificial cerebrospinal fluid was perfused and collected to measure lactate. The extracellular lactate levels in the dialysate samples were determined using a lactate assay kit. Control samples were collected from naive WT mice. The extracellular lactate levels during severe middle cerebral artery occlusion (MCAO; for 1 h) were increased compared with the control. In mice who received preconditioning (PC; 15 min of MCAO) 3 d before severe MCAO, extracellular lactate levels during severe MCAO had a greater increase compared with mice who underwent severe MCAO without PC. When P2X7 receptor knock-out mice received PC 3 d before severe MCAO, their extracellular lactate levels during severe MCAO were lower than those in WT mice. Data are presented as the fold increase over control (naive WT) mice. Values are shown as mean ± SEM; **p < 0.01, one-way ANOVA followed by Tukey's post hoc multiple-comparisons test; *n* = 4 (***a***); *p < 0.05, unpaired two-tailed Student's *t* test; *n* = 3–4 (***b***). Additional data are presented in Extended Data Figure 1-1. ***c***, Mice received a lateral ventricle injection of 1 µl of 100 mM lactate 1 d before severe MCAO. Brain damage was assessed by TTC staining of coronal brain sections. Lactate injection protected against severe MCAO-induced ischemic injury. Scale bar, 2 mm. The results are summarized in ***d***. Values are shown as mean ± SEM; **p < 0.01, unpaired two-tailed Student's *t* test; *n* = 5–6.

For the data shown in Figure 1, ʟ-lactate (Sigma-Aldrich) was diluted to a final concentration of 100 mM in phosphate-buffered saline (PBS), pH 7.4. The lactate solution was stereotaxically microinjected into the lateral ventricle of the brain (anterior, −0.7 mm; lateral, 1.5 mm from bregma; depth, 2 mm from the skull surface) 1 d before 1 h of MCAO. For this injection, 1 µl of 100 mM lactate solution was injected continuously at a rate of 0.5 µl/min through a microinjection cannula (AMI-2, Eicom) connected to a 10 µl syringe. For control mice, the same volume of PBS, pH 7.4, was injected into the lateral ventricle of the brain. To investigate whether CD147 is required for PC-induced ischemic tolerance, 2 d after PC, anti-CD147 (clone: RL73; eBioscience) or its isotype control (clone: eBR2a; eBioscience) was stereotaxically microinjected into the lateral ventricle of the brain as follows: 1 µl of anti-CD147 (1 mg/ml) or its isotype control (1 mg/ml) was injected continuously at a rate of 0.5 µl/min through a microinjection cannula connected to a 10 µl syringe. To investigate the role of P2X7 receptors in lactate release during severe ischemia, the P2X7 receptor antagonist JNJ-47965567 (30 mg/kg; Selleck Chemicals) dissolved in 30% sulfobutylether-β-cyclodextrin solution was injected intraperitoneally into mice 1 d before severe MCAO (i.e., 1 h of occlusion) was induced. For control mice, the same volume of 30% sulfobutylether-β-cyclodextrin solution was injected intraperitoneally.

10.1523/ENEURO.0494-23.2024.f1-1 Figure 1-1 **The P2X7 receptor antagonist JNJ-47965567 suppressed the preconditioning (PC)-induced enhancement of increased extracellular lactate levels during severe ischemia.** JNJ-47965567 (30  mg/kg) was intraperitoneally injected 1 day before severe middle cerebral artery occlusion (MCAO; for 1  h). Although intraperitoneal JNJ-47965567 administration had no effects on extracellular lactate levels during severe MCAO alone (i.e., without PC [15  min of MCAO]), this treatment suppressed the PC-induced enhancement of the increase in extracellular lactate levels during severe MCAO. Data show the fold increase over control (naïve) mice. Values are shown as means ± SEM; *P < 0.05, two-way ANOVA followed by Tukey’s post hoc multiple comparison test; n = 3–4. NS, not significant. Download Figure 1-1, TIF file.

### Analysis of cerebral infarct size and edema

Mice were anesthetized with 4% (v/v) isoflurane and perfused transcardially with saline. The brains were removed, and the forebrain was then sliced into 2-mm-thick coronal sections using Rodent Brain Matrices (ASI Instruments). The sections were stained with 2% [weight/volume (w/v)] 2,3,5-triphenyltetrazolium chloride (TTC; Sigma-Aldrich) saline solution at 37°C for 10 min. After staining, slices were digitally scanned using a scanner (GT-X830, Epson). The infarct volume and edema of each brain slice were measured using Scion Image software (Scion). Infarct size was calculated as the infarct volume of the whole brain or striatum / volume of the whole brain × 100. The edema rate of the striatum (anterior, 0.14 mm from bregma), which is an area served by the MCA, was calculated as follows: (area of the ipsilateral striatum − area of the contralateral striatum) / area of the contralateral striatum × 100.

### Measurement of extracellular lactate concentration

The mice were anesthetized with 4% (v/v) isoflurane, maintained on 2% (v/v) isoflurane, and placed in a stereotaxic instrument (Narishige) before the skull bone was exposed. The bone surface was sterilized with 70% ethanol and povidone-iodine, and a hole was drilled with a 1.0 mm bit on a high-speed drill (MINITOR UC210, URAWA Kogyo). Through this hole, a microdialysis guide cannula (AG-2, Eicom) was implanted in the right striatum (anterior, 0 mm; lateral, 2.5 mm from bregma; depth, 2 mm from the skull surface). The external portion of the cannula was fixed to the skull using an anchor screw and dental cement (ESTECEM II, Tokuyama Dental). A dummy probe (AD-2, Eicom) was then inserted into the right striatum of freely moving mice via the guide cannula and fixed with a cap nut (AC-5, Eicom). Mice were allowed 1 d of recovery before the dummy probe was exchanged for a microdialysis probe (A-I-2-01, cut off: MW50,000, Eicom). Probes were attached to microbore tubing that traveled through a microdialysis swivel (TCS2-23, Eicom) and head tether assembly (WT-20 T, Eicom), which allowed the animal to move around the cage. In vivo microdialysis was then performed on conscious, freely moving mice. Using a microperfusion pump (KD Scientific), the microdialysis probes were perfused with artificial cerebrospinal fluid (composition in mM as follows: 152 NaCl, 2.5 KCl, 2 CaCl_2_, 1 MgCl_2_, 10 HEPES, pH 7.4) at a constant flow rate of 3 µl/min. The equilibration time was 30 min. Focal cerebral ischemia was induced by MCAO in the right hemisphere. After the operation, the mice were returned to the microdialysis system, and samples were collected for 1 h. Extracellular lactate levels in the samples were measured using a lactate assay kit (BioVision) and a microplate reader (Multiskan FC, Thermo Fisher Scientific K.K.).

### Quantification of glycogen

Mice were anesthetized with 4% (v/v) isoflurane and perfused transcardially with saline. The brains were removed, and the forebrain was then sliced into 2-mm-thick coronal sections using Rodent Brain Matrices. After trimming, ipsilateral striatal sections were homogenized, and glycogen concentrations were measured using a glycogen assay kit (BioVision) and a microplate reader.

### Immunohistochemistry

Mice were anesthetized with 4% (v/v) isoflurane and perfused transcardially with saline, followed by 4% (w/v) paraformaldehyde in PBS. The brains were removed, postfixed overnight in a solution containing 4% (w/v) paraformaldehyde and 4% (w/v) sucrose in PBS, and cryoprotected in solutions containing 10% (w/v) and 20% (w/v) sucrose in PBS for 1 d each. The brains were then frozen in an embedding compound (Sakura Finetek) on dry ice before coronal sections (20 µm) were cut on a cryostat (CM 1100, Leica). The sections were fixed with 4% (w/v) paraformaldehyde for 30 min, permeabilized with 0.3% (v/v) Triton X-100 for 20 min, and treated with 3% (w/v) bovine serum albumin in PBS for 30 min to block nonspecific binding. Next, the sections were incubated for 3 d at 4°C with the following primary antibodies: rabbit anti-glial fibrillary acidic protein (GFAP; 1:1,000; Millipore), mouse anti-MCT1 (1:250; Abcam), mouse anti-MCT4 (1:50; Santa Cruz Biotechnology), or mouse anti-CD147 (1:50; Santa Cruz Biotechnology). After being washed, the sections were incubated for 1 h at room temperature with the following secondary antibodies: Alexa 488- or Alexa 546-conjugated anti-mouse or -rabbit IgG (Invitrogen). Fluorescence images were obtained using a confocal laser scanning microscope (LSM 780, Carl Zeiss, Jena, Germany).

### Astrocyte cultures

Primary cultures of astrocytes were prepared from cerebral cortices of postnatal day 0 C57BL/6J mice. Dissected cerebral cortices were dissociated in Dulbecco's PBS (Invitrogen, Life Technologies). The cells were then plated in six-well poly-ʟ-lysine–coated cell culture plates (two hemispheres/plate) in Dulbecco's modified Eagle's medium (Wako) containing 10% (v/v) fetal bovine serum and 1% (v/v) penicillin/streptomycin. Upon reaching confluence, the astrocyte cultures were treated with 8 µM cytosine β-D-arabinofuranoside (Ara-C; Sigma-Aldrich) for 6–7 d to prevent microglial proliferation. This treatment is not cytotoxic to quiescent astrocytes (Hamby et al., 2006). The cells were grown, maintained, and stimulated at 37°C in a humidified atmosphere of 5% CO_2_. All studies were performed between 13 and 15 d in vitro. Cells were treated with BzATP (Sigma-Aldrich) for 24 h.

### Western blot analysis

Cultured astrocytes were prepared as described above, Astrocyte cultures. After washing with Dulbecco's PBS, the cells were lysed. The lysates were then resolved on a 12.5% (w/v) sodium dodecyl sulfate polyacrylamide gel and transferred to polyvinylidene fluoride membranes. Next, the membranes were blocked for 1 h at room temperature in Tris-buffered saline containing 0.1% (v/v) Tween 20 (TBS-T) and 4% (w/v) skim milk before being incubated overnight at 4°C with the following primary antibodies: mouse anti-HIF-1α (1:400; Novus Biologicals), rabbit anti-MCT1 (1:400; Novus Biologicals), rabbit anti-MCT4 (1:300; Novus Biologicals), mouse anti-CD147 (1:100; Santa Cruz Biotechnology), mouse anti-Na^+^/K^+^-ATPase α1(1:1,000; Santa Cruz Biotechnology), or mouse anti-β-actin (1:10,000; Sigma-Aldrich). After three washes with TBS-T, the membranes were incubated for 1 h at room temperature with horseradish peroxidase-conjugated anti-mouse antibody (1:10,000; GE HealthCare) or anti-rabbit antibody (1:10,000; GE HealthCare). The membranes were then washed three times with TBS-T, and the proteins were visualized using the Chemi-Lumi One Ultra system (Nacalai Tesque). Images were obtained using an LAS-4000 imager (Fujifilm).

### Immunocytochemistry

Cultured astrocytes were prepared as described above, Astrocyte cultures. The cells were plated in a 35-mm µ-Dish (ibidi) and treated with 10 µM BzATP for 24 h. The cultured astrocytes were then fixed with 4% (w/v) paraformaldehyde for 30 min, permeabilized with 0.3% (v/v) Triton X-100 for 10 min, and treated with 3% (w/v) bovine serum albumin in PBS for 30 min to block nonspecific binding. Next, the cells were incubated for 2 d at 4°C with the following primary antibodies: rabbit anti-MCT1 (1:300; Novus Biologicals) or mouse anti-MCT4 (1:50; Santa Cruz Biotechnology). The cells were washed before being incubated for 1 h at room temperature with the following secondary antibodies: Alexa 546-conjugated anti-mouse or anti-rabbit IgG (Invitrogen). The nuclei were then counterstained with 4′,6-diamidino-2-phenylindole solution (Nacalai Tesque). Fluorescence images were obtained using a confocal laser scanning microscope.

### Membrane protein extraction

Cultured astrocytes were prepared as described above, Astrocyte cultures. The cells were plated in a 100 mm dish before being treated with 10 µM BzATP and 0.1 µg/ml anti-CD147 or its isotype control for 24 h. Membrane proteins in the astrocytes were then extracted using a Mem-PER Plus Membrane Protein Extraction Kit (Thermo Fisher Scientific).

### Experimental design and statistical analysis

All statistical tests were conducted using Prism 7 (GraphPad Software). Data are presented as the mean ± standard error of the mean (SEM) with raw dot plots. Statistical analyses were performed using an unpaired two-tailed Student's *t* test, unpaired two-tailed Welch's *t* test, one-way analysis of variance (ANOVA) followed by Tukey's or Dunnett's post hoc multiple-comparisons test, or two-way ANOVA followed by Tukey's post hoc multiple-comparisons test. Details of the statistical tests are indicated in the figure legends. *p* < 0.05 was considered statistically significant.

## Results

### P2X7 receptors regulate extracellular lactate levels in a PC-induced ischemic tolerance model

Although extracellular lactate levels are increased by cerebral ischemia (Hillered et al., 1989), the effect of PC (15 min of MCAO) on extracellular lactate levels during severe MCAO (1 h of MCAO) remains unknown. Using an MCAO mouse model combined with a microdialysis technique, we measured extracellular lactate levels by inserting a probe into the ipsilateral striatum. In this brain region, astrocytes are activated and upregulate P2X7 receptors 3 d after PC, and PC-induced ischemic tolerance is observed in wild-type (WT) but not P2X7^−/−^ mice (Hirayama et al., 2015). In the present study, we first confirmed that extracellular lactate levels were increased by severe MCAO (Fig. 1*a*). Moreover, when mice received PC 3 d before severe MCAO, the severe MCAO-induced increase in extracellular lactate levels was further enhanced. In contrast, when P2X7^−/−^ mice received PC 3 d before severe MCAO, the extracellular lactate levels during severe MCAO were lower than those of WT mice (Fig. 1*b*). Furthermore, although the intraperitoneal administration of a centrally permeable P2X7 receptor antagonist JNJ-47965567 (Bhattacharya et al., 2013) had no effect on extracellular lactate levels during severe MCAO alone (i.e., without PC), this treatment suppressed the PC-induced enhancement of the increase in extracellular lactate levels during severe MCAO (Extended Data Fig. 1-1). Together, these results suggest that astrocytic P2X7 receptors regulate lactate release into the extracellular space in the PC-induced ischemic tolerance model.

### Lactate is neuroprotective against cerebral ischemic injury

Using an MCAO mouse model, we investigated the effects of lactate on cerebral ischemic injury. The intracerebroventricular injection of lactate protected against cerebral ischemic injury (Fig. 1*c*,*d*). This finding raises the possibility that extracellular lactate plays a crucial role in PC-induced ischemic tolerance.

### PC-evoked glycogen accumulation occurs independently of P2X7 receptors

**Figure 2.**
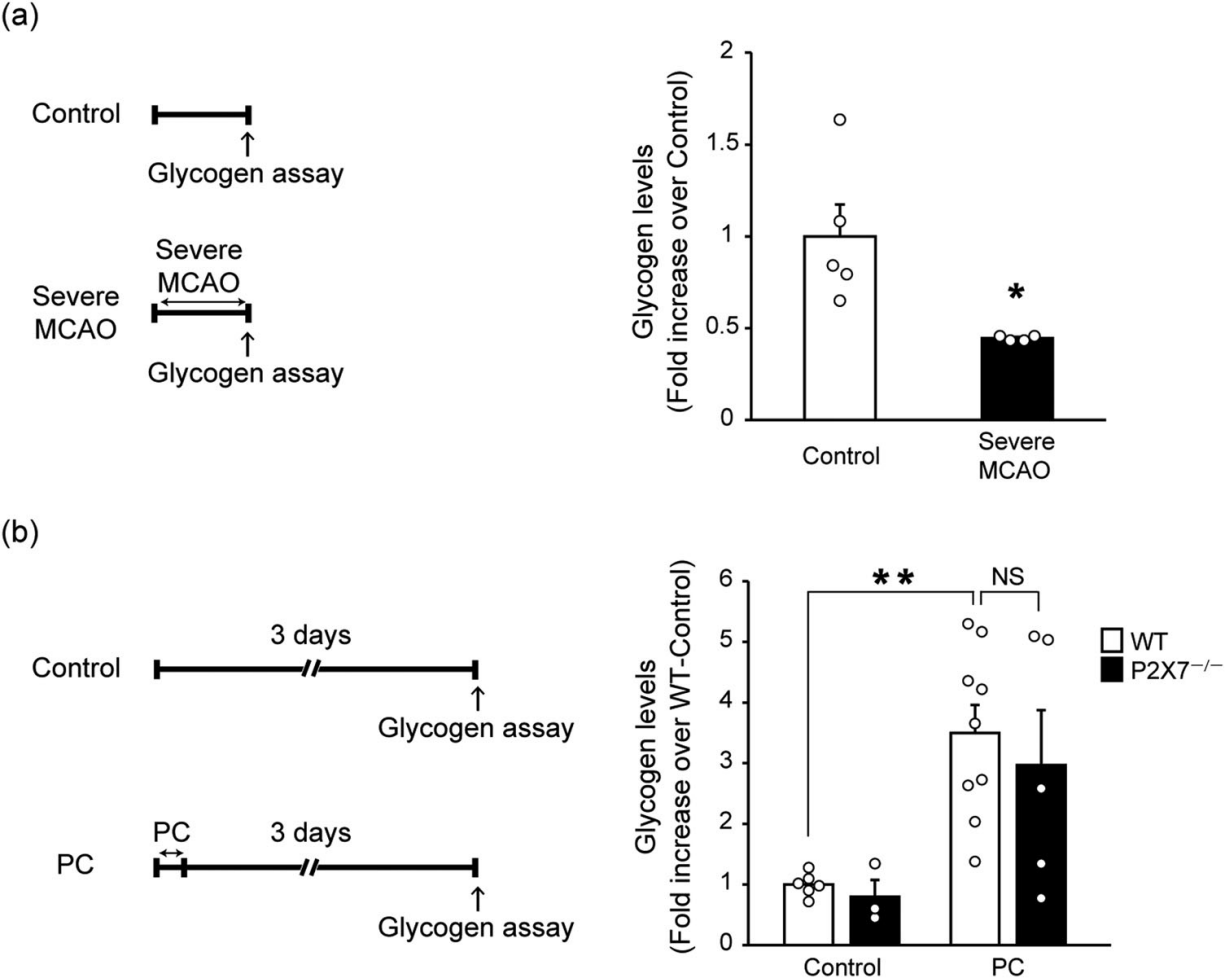
Preconditioning (PC)-evoked and P2X7 receptor-independent glycogen accumulation. ***a***, Glycogen levels in the ipsilateral striatum were determined using a glycogen assay kit. Control samples were collected from sham-operated control mice. Glycogen levels after severe middle cerebral artery occlusion (MCAO; for 1 h) were decreased. Values are shown as mean ± SEM; *p < 0.05, unpaired two-tailed Welch's *t* test; *n* = 4–5. ***b***, Although glycogen levels in the ipsilateral striatum were significantly increased 3 d after PC (15 min of MCAO), there was no difference in PC-evoked glycogen accumulation between WT and P2X7 receptor knock-out mice. Values are shown as mean ± SEM; **p < 0.01, two-way ANOVA followed by Tukey's post hoc multiple-comparisons test; *n* = 3–9. NS, not significant.

Next, we investigated the possible mechanisms of the PC-induced P2X7 receptor-mediated increase in extracellular lactate levels during severe MCAO. In the brain, glycogen—a source of lactate that is degraded to lactate during ischemia—is stored predominantly in astrocytes (Oe et al., 2016). In addition, HIF-1α controls the expression of genes that encode the enzymes required to convert glucose to glycogen (Semenza, 2013). We therefore examined the possibility that PC might increase astrocytic glycogen via the P2X7 receptor/HIF-1α signaling pathway, thus leading to the facilitation of lactate production and release during severe MCAO. Cerebral ischemia reportedly decreases glycogen levels (Cai et al., 2020); we confirmed this using a glycogen assay kit (Fig. 2*a*). Notably, glycogen levels in the ipsilateral striatum were increased 3 d after PC (Fig. 2*b*). However, there was no significant difference in glycogen levels between WT and P2X7^−/−^ mice. These results suggest that PC-facilitated glycogen accumulation is not involved in the P2X7 receptor-mediated increase in extracellular lactate levels during severe MCAO.

### Molecules related to lactate release are increased in astrocytes after PC

**Figure 3.**
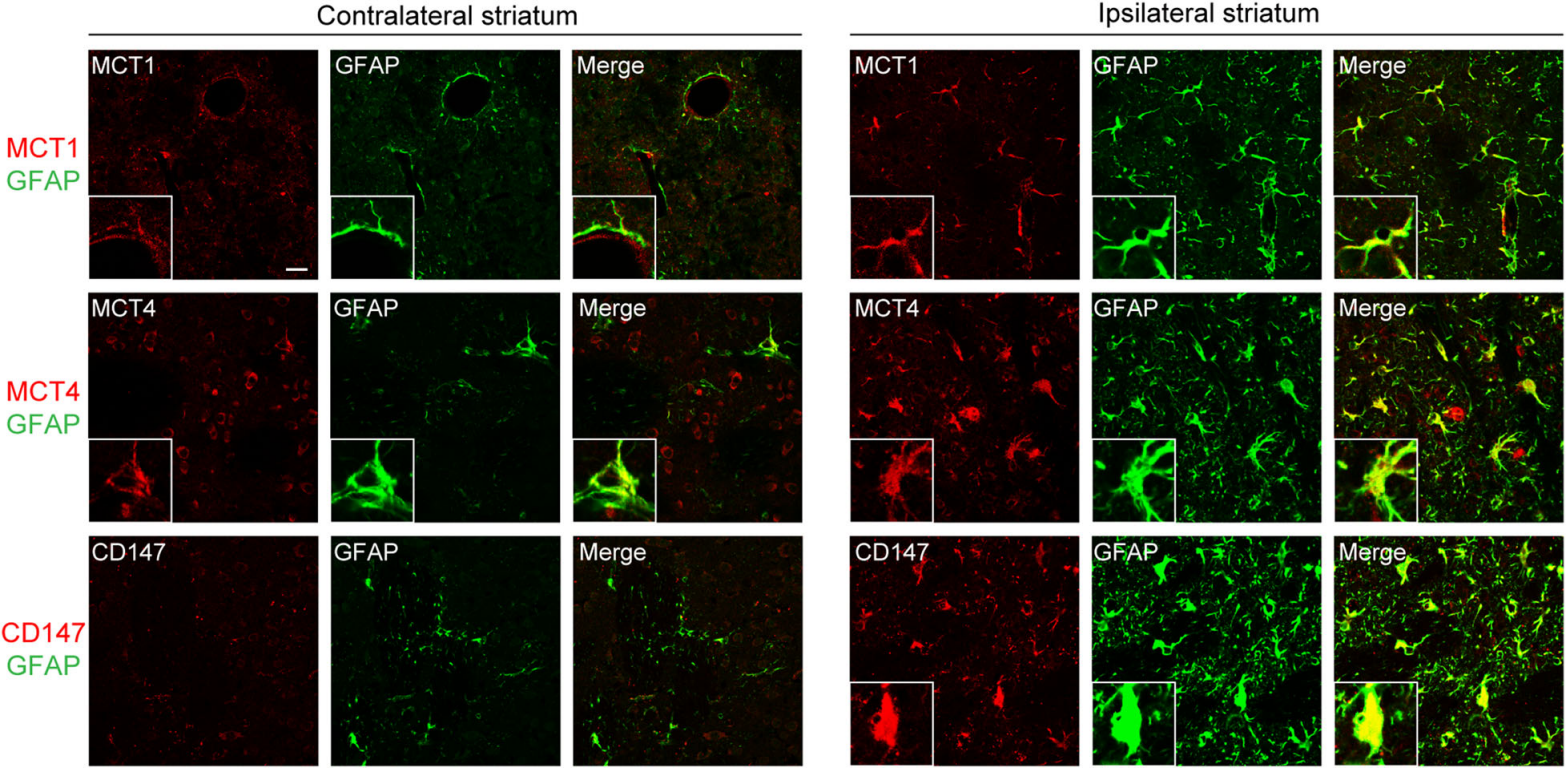
Upregulation of molecules related to lactate release in astrocytes after preconditioning (PC). Representative immunohistochemical images of the striatum stained with anti-glial fibrillary acidic protein (GFAP; green) and anti-monocarboxylate transporter (MCT) 1 (red), anti-MCT4 (red), or anti-CD147 (red) antibodies. Three days after PC (15 min of middle cerebral artery occlusion), MCT1 and MCT4, but not CD147, were expressed in GFAP-positive astrocytes in the contralateral striatum. Compared with those in the contralateral striatum, astrocytes in the ipsilateral striatum had markedly increased MCT1, MCT4, and CD147. Scale bars: main images, 20 µm; insets, 8 µm.

In this experiment, we examined whether PC increases molecules related to lactate release in astrocytes via the P2X7 receptor/HIF-1α signaling pathway, thus leading to increased extracellular lactate levels during severe MCAO. In the brain, astrocytes express MCT1 and MCT4, which are essential for the release of lactate into the extracellular space (Pierre and Pellerin, 2005). In addition, CD147—a chaperone of MCT1 and MCT4—assists in the membrane translocation of these MCTs (Kirk et al., 2000). To investigate the expression patterns of these molecules after PC, we performed double immunohistochemical staining. Three days after PC, MCT1 and MCT4 were expressed in GFAP-positive astrocytes in the contralateral striatum (Fig. 3). In the ipsilateral striatum, MCT1 and MCT4 expression in astrocytes was markedly increased compared with the contralateral striatum. Although CD147-positive signals were mostly absent in the contralateral striatum, they were increased, mainly in astrocytes, in the ipsilateral striatum. This finding suggests that the induction of these molecules in astrocytes may be responsible for the PC-induced increase in extracellular lactate levels during severe MCAO.

### Expression of CD147 in astrocytes is dependent on P2X7 receptors

**Figure 4.**
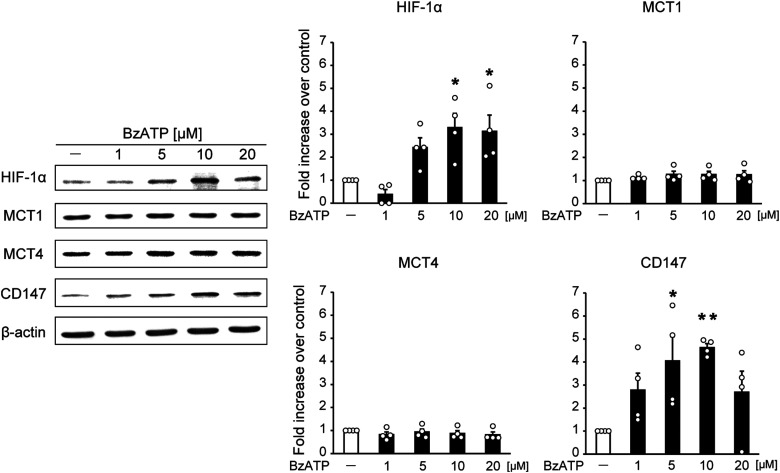
P2X7 receptor agonist treatment upregulated CD147 in cultured astrocytes. Primary astrocyte cultures were treated with various concentrations of the P2X7 receptor agonist BzATP for 24 h at the indicated concentrations. Western blotting was then performed. Protein levels of HIF-1α and its target gene CD147 were significantly increased by BzATP treatment. In contrast, monocarboxylate transporter (MCT) 1 and MCT4 protein levels were unchanged by BzATP treatment. Data are representative of four independent experiments. The levels of these proteins were normalized to those of β-actin. Data show the fold increase over controls (no treatment). Values are shown as mean ± SEM; *p < 0.05, **p < 0.01 versus control, one-way ANOVA followed by Dunnett's post hoc multiple-comparisons test; *n* = 4.

Although PC induced the astrocytic upregulation of MCT1, MCT4, and CD147, it remained unclear whether the expression of these molecules was dependent on P2X7 receptor signaling. We next investigated the effects of the P2X7 receptor agonist BzATP on MCT1, MCT4, and CD147 expression in cultured astrocytes, which express P2X7 receptors (Panenka et al., 2001; Hirayama et al., 2015). We confirmed that BzATP treatment in cultured astrocytes led to increased HIF-1α, a downstream target of P2X7 receptors (Fig. 4). Furthermore, CD147, but not MCT1 or MCT4, was significantly increased by BzATP treatment. Together, these results suggest that the PC-evoked upregulation of CD147 is dependent on P2X7 receptors in astrocytes.

### Activation of P2X7 receptors results in the plasma membrane translocation of MCT1 and MCT4 via CD147 in astrocytes

**Figure 5.**
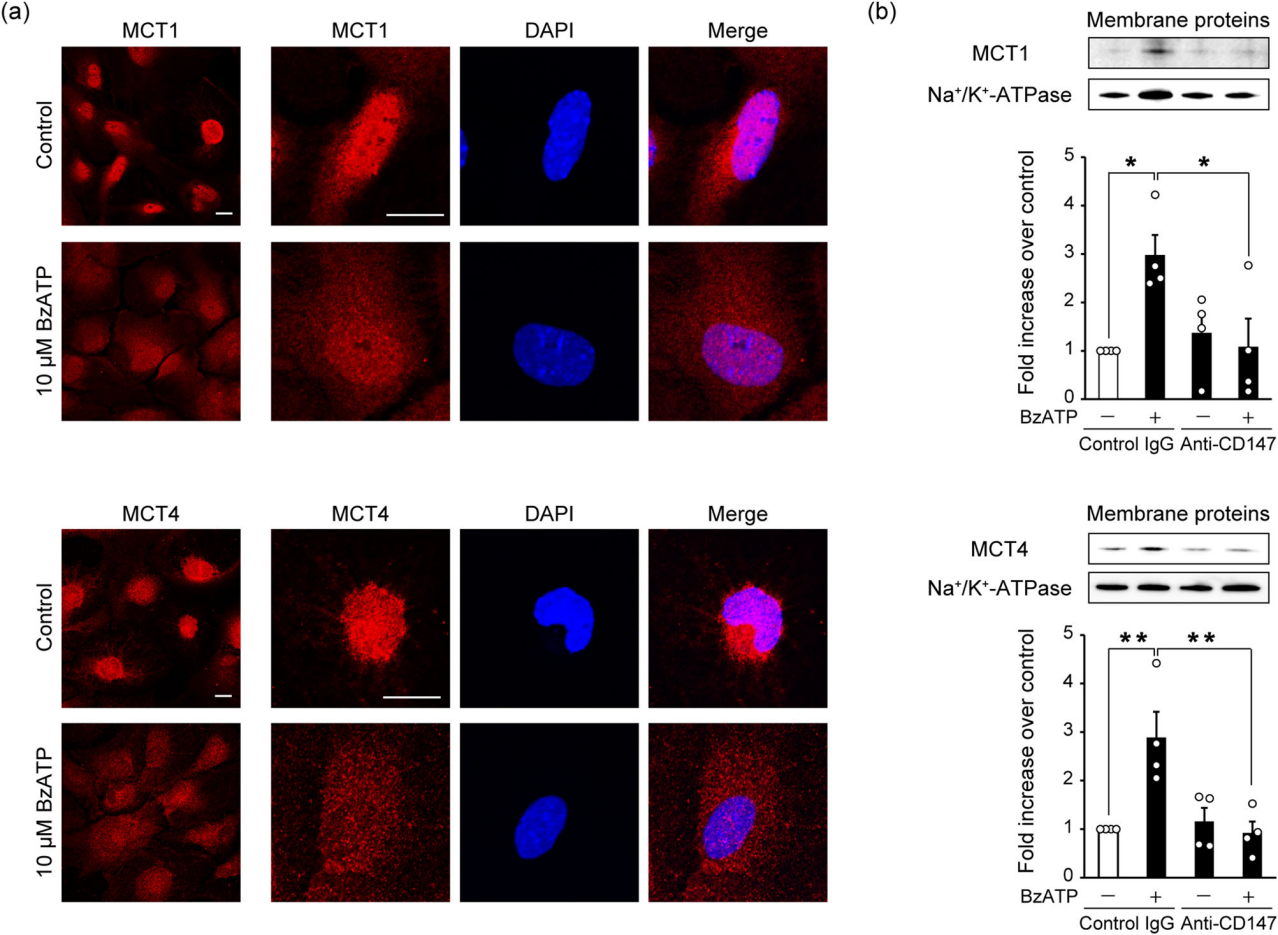
P2X7 receptor agonist evoked the membrane translocation of monocarboxylate transporter (MCT) 1 and MCT4 via CD147 in cultured astrocytes. ***a***, Primary astrocyte cultures were treated with 10 µM of the P2X7 receptor agonist BzATP for 24 h. Immunocytochemistry was then performed. Representative immunocytochemical images of the cultured astrocytes stained with anti-MCT1 (red) or anti-MCT4 (red) antibodies are shown. BzATP treatment induced the translocation of MCT1 and MCT4 from the perinuclear region to the cell surface in cultured astrocytes. Scale bar, 20 µm. ***b***, Primary astrocyte cultures were treated with 10 µM BzATP and 0.1 µg/ml anti-CD147 or its isotype control for 24 h. Membrane protein extraction and Western blotting were then performed. In cultured astrocytes, the membrane expression levels of MCT1 and MCT4 were significantly increased by BzATP treatment; however, these increases were suppressed by anti-CD147. Data are representative of four independent experiments. The levels of these proteins were normalized to those of Na^+^/K^+^-ATPase. Data show the fold increase over control (control IgG treatment). Values are shown as mean ± SEM; *p < 0.05, **p < 0.01, two-way ANOVA followed by Tukey's post hoc multiple-comparisons test; *n* = 4.

To investigate any changes in the localization of MCT1 and MCT4 caused by the P2X7 receptor agonist BzATP, we performed immunocytochemical staining in cultured astrocytes. The activation of P2X7 receptors by BzATP induced the translocation of MCT1 and MCT4 from the perinuclear region to the cell surface (Fig. 5*a*). In addition, the membrane expression of MCT1 and MCT4 was significantly increased by BzATP treatment, and these increases were suppressed by CD147-blocking antibody (Fig. 5*b*). These findings suggest that P2X7 receptor activation promotes the membrane expression of MCT1 and MCT4 via CD147 without changing the total expression levels of these transporters.

### Lactate release from astrocytes via CD147 is essential for PC-induced ischemic tolerance

**Figure 6.**
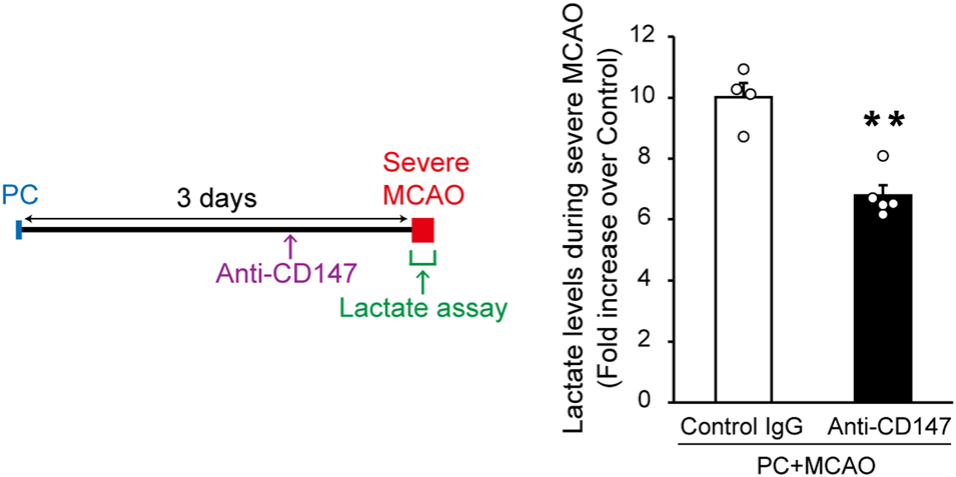
Preconditioning (PC)-evoked and CD147-mediated increase in extracellular lactate levels during severe ischemia. One microliter of anti-CD147 (1 mg/ml) or its isotype control (1 mg/ml) was stereotaxically microinjected into the lateral ventricle of the brain 2 d after PC [15 min of middle cerebral artery occlusion (MCAO)]. The PC-evoked increase in extracellular lactate levels during severe MCAO (1 h of MCAO) was suppressed by anti-CD147. Data show the fold increase over control (naive) mice. Values are shown as mean ± SEM; **p < 0.01, unpaired two-tailed Student's *t* test; *n* = 4–5.

**Figure 7.**
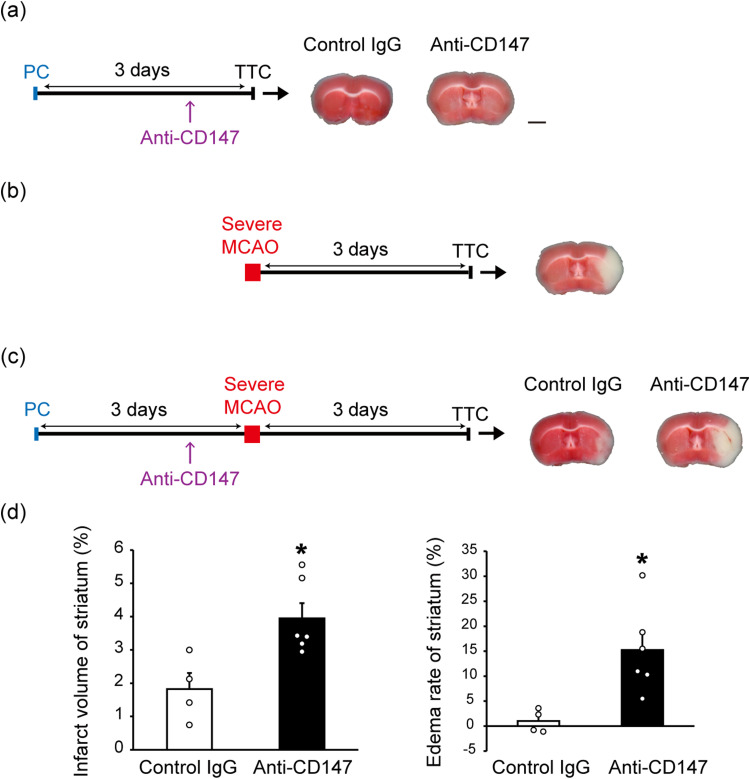
Inhibition of preconditioning (PC)-induced ischemic tolerance by CD147-blocking antibody. ***a***, One microliter of anti-CD147 (1 mg/ml) or its isotype control (1 mg/ml) was stereotaxically microinjected into the lateral ventricle of the brain 2 d after PC [15 min of middle cerebral artery occlusion (MCAO)]. PC caused no damage. Furthermore, anti-CD147 injection after PC had no effect on TTC staining. Scale bar, 2 mm. ***b***, One hour of MCAO-induced severe injury (mainly in the striatum and cortex) and was used to assess MCAO-induced brain injury (termed severe MCAO). ***c***, Severe MCAO-evoked damage in the striatum was significantly reduced when mice received PC 3 days before experiencing severe MCAO. However, this ischemic tolerance was almost abolished when mice were injected with anti-CD147. The results are summarized in ***d***. Values are shown as mean ± SEM; *p < 0.05, unpaired two-tailed Student's *t* test; *n* = 4–6.

To investigate whether CD147 is required for PC-induced ischemic tolerance, we investigated the effects of CD147-blocking antibody on ischemic tolerance. Anti-CD147 was injected into the lateral ventricle of the brain 2 d after PC to inhibit CD147 function. This anti-CD147 injection suppressed the PC-induced increase in extracellular lactate levels during severe MCAO (Fig. 6). Notably, anti-CD147 injection abolished PC-induced ischemic tolerance (Fig. 7); that is, PC-induced decreases in infarct size and edema were no longer detected. Furthermore, the injection of anti-CD147 itself did not produce brain damage (Fig. 7*a*). Together, these results suggest that CD147 plays a crucial role in PC-induced ischemic tolerance.

## Discussion

**Figure 8.**
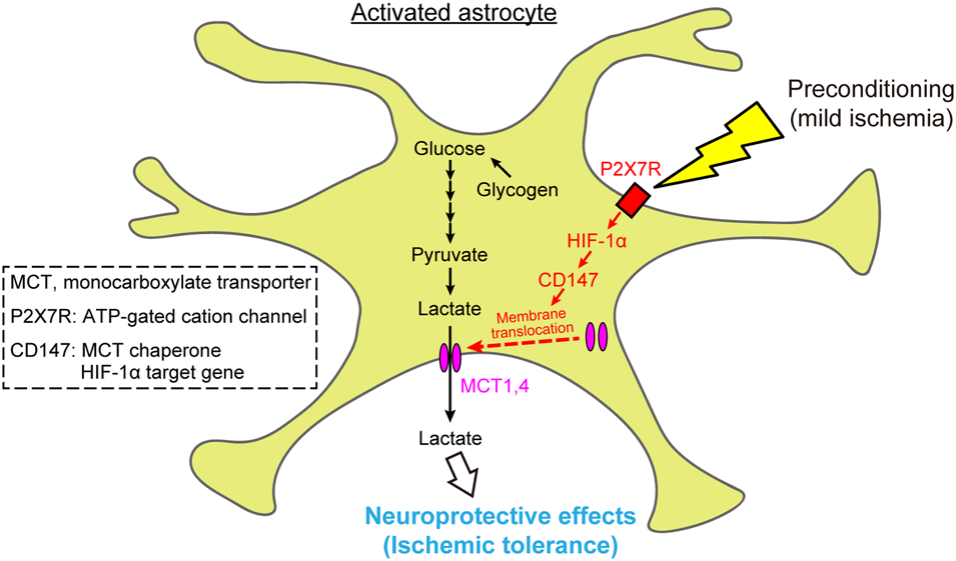
Schematic diagram of the mechanisms underlying preconditioning (PC)-induced ischemic tolerance. PC-evoked astrocytic activation induces CD147 expression via the P2X7 receptor/HIF-1α signaling pathway. This CD147 expression assists with the translocation of monocarboxylate transporter (MCT) 1 and MCT4 to the cell membrane, thereby promoting lactate release from astrocytes during severe ischemia; this effect likely plays a role in ischemic tolerance.

We have previously reported that PC-evoked astrocytic activation induces ischemic tolerance via the P2X7 receptor/HIF-1α signaling pathway (Hirayama et al., 2015). However, the downstream mechanisms of this pathway remain unknown. Here, we demonstrated that the PC-induced activation of the P2X7 receptor/HIF-1α signaling pathway in astrocytes leads to CD147 expression. This expression of CD147 helps to guide MCT1 and MCT4 to the cell membrane, thereby promoting lactate release from astrocytes during severe ischemia, which likely plays a role in ischemic tolerance (Fig. 8).

After Pellerin and Magistretti proposed the hypothesis of an astrocyte–neuron lactate shuttle (Pellerin and Magistretti, 1994), the various roles of lactate released from astrocytes have been revealed. For example, Suzuki et al. (2011) demonstrated that lactate transport from astrocytes to neurons via MCTs is required for long-term memory formation (Suzuki et al., 2011). Moreover, in pathological conditions, seizures can reportedly be reduced by blocking lactate dehydrogenase, which is a component of the astrocyte–neuron lactate shuttle (Sada et al., 2015). Lactate has traditionally been considered a toxic waste product because its intracellular accumulation leads to acidosis. In cerebral ischemia, lactic acidosis in astrocytes causes edema by increasing water permeability via aquaporin 4, the principal water channel in the brain (Vella et al., 2015), and excess glutamate release, thus leading to excitotoxicity (Beppu et al., 2014). The facilitation of lactate release from astrocytes during severe ischemia may therefore prevent intracellular acidosis, leading to protection. Furthermore, extracellular lactate serves as a neuronal energy source (Wyss et al., 2011) and has functions that include reducing neuronal excitability via the lactate receptor HCA1 (Abrantes et al., 2019) and increasing vasodilation by attenuating prostaglandin transporter efficacy (Gordon et al., 2008), both of which are associated with protection against cerebral ischemic injury. In the present study, PC-facilitated lactate release from astrocytes during severe ischemia in a P2X7 receptor-dependent manner (Fig. 1*a*,*b*) and may therefore be responsible for ischemic tolerance. However, further investigations are required to confirm this possibility.

In the brain, P2X7 receptor–lactate interactions remain relatively poorly understood. P2X7 receptors are nonselective cation channels that are activated by extracellular adenosine triphosphate; they contribute to many central nervous system diseases (Matute et al., 2007; Hirayama et al., 2022). Although P2X7 receptors control various pathophysiological events, including proliferation (Adinolfi et al., 2012), phagocytosis (Leeson et al., 2018), and inflammatory responses (Chakfe et al., 2002; Iwata et al., 2016), their involvement in lactate metabolism and transport remains unknown. To elucidate the mechanisms of the PC-evoked, P2X7 receptor-mediated increase in extracellular lactate levels during severe ischemia, we focused on glycogen in the brain. We investigated whether PC increases glycogen in a P2X7 receptor-dependent manner, leading to the aforementioned event. Glycogen is a major source of lactate in cerebral ischemia and is predominantly stored in astrocytes (Oe et al., 2016). Although PC-evoked glycogen accumulation was observed in the present study, glycogen levels after PC did not differ between WT and P2X7^−/−^ mice (Fig. 2*b*). We therefore concluded that PC-evoked glycogen accumulation is not involved in the P2X7 receptor-mediated increase in extracellular lactate levels during severe ischemia.

Next, we investigated PC-induced changes in lactate transport. Among the 14 MCT isoforms (MCT1–14), MCT1, MCT2, and MCT4 are expressed in the brain and reportedly catalyze the proton-coupled transport of lactate (Bergersen, 2015). Neurons express predominantly MCT2, which regulates lactate influx, whereas astrocytes express MCT1 and MCT4, which serve as lactate exporters during ischemia (Zhang et al., 2022). In the current study, we speculated that PC may increase MCT1 and MCT4 in astrocytes in a P2X7 receptor-dependent manner to facilitate lactate release during severe ischemia. Although PC increased MCT1 and MCT4 in astrocytes, these increases were independent of P2X7 receptors (Figs. 3, 4). Astrocytes also had increased CD147 expression after PC, and this expression was dependent on P2X7 receptors.

CD147 (also known as extracellular matrix metalloproteinase inducer or basigin) is a transmembrane glycoprotein that is a target gene of HIF-1α (Ke et al., 2012). CD147 is an important upstream regulator of matrix metalloproteinases, which are associated with tumor progression and invasion (Kanekura and Chen, 2010). Recent studies have shown that, in cerebral ischemia, CD147 induces the production of matrix metalloproteinase 9 (MMP-9). This contributes to secondary damage after ischemia via the breakdown of the blood–brain barrier and the recruitment of peripheral leukocytes into the brain; the administration of CD147-blocking antibody after ischemia thus leads to neuroprotection (Jin et al., 2017; Patrizz et al., 2020). Interestingly, the present results indicated some unexpected roles of CD147 in cerebral ischemia. CD147 is a chaperone of MCT1 and MCT4 and assists with their translocation to the cell membrane (Kirk et al., 2000). In the current study, activation of the P2X7 receptor/HIF-1α signaling pathway in astrocytes increased CD147 expression and promoted the membrane translocation of MCT1 and MCT4 via CD147 (Figs. 4, 5). Furthermore, CD147 was responsible for the PC-induced increase in extracellular lactate levels during severe ischemia, leading to ischemic tolerance (Figs. 6, 7). We therefore speculate that the role of CD147-blocking antibody varies with the timing of treatment relative to cerebral ischemia. Specifically, it may be that the administration of CD147-blocking antibody before ischemia has a deleterious effect by suppressing the PC-induced facilitation of lactate release from astrocytes, whereas its administration after ischemia provides neuroprotection by inhibiting MMP-9 production. Although CD147 is essential for PC-induced brain ischemic tolerance, it remains unclear whether the PC-evoked, P2X7 receptor-independent upregulation of MCT1 and MCT4 in astrocytes is necessary. In addition, recent studies indicate that MCT1 and MCT4 have different roles. For example, MCT1, but not MCT4, mediates lactate efflux in glioma cells (Miranda-Goncalves et al., 2016). In the present study, any differences in roles between MCT1 and MCT4 in PC-induced ischemic tolerance remain unknown and need to be clarified in future studies.

In conclusion, we demonstrated that the PC-evoked and P2X7 receptor/HIF-1α signaling pathway-dependent upregulation of CD147 in astrocytes promotes the membrane translocation of MCT1 and MCT4. This translocation leads to increased extracellular lactate levels during severe ischemia, followed by the induction of ischemic tolerance. Our results also suggest that CD147 is a key molecule that regulates lactate release from astrocytes, thus mediating neuroprotection. This insight may facilitate the development of new therapeutic strategies for cerebral ischemia.

## Data Availability

The data that support the findings of this study are available from the corresponding author upon reasonable request.

## Synthesis

Reviewing Editor: Masha Prager-Khoutorsky, McGill University Decisions are customarily a result of the Reviewing Editor and the peer reviewers coming together and discussing their recommendations until a consensus is reached. When revisions are invited, a fact-based synthesis statement explaining their decision and outlining what is needed to prepare a revision will be listed below. The following reviewer(s) agreed to reveal their identity: Masabumi Minami. Note: If this manuscript was transferred from JNeurosci and a decision was made to accept the manuscript without peer review, a brief statement to this effect will instead be what is listed below.

Rev#1

In this study, the authors extended their previous work in which they revealed the crucial roles of P2X7 receptors in ischemic tolerance by showing that activation of P2X7 receptors during sub-lethal ischemic episode (preconditioning) enhanced the astrocytic lactate release during subsequent severe ischemia thereby inducing ischemic tolerance. They also demonstrated that They also showed that such enhancement of lactate release is due to increased translocation of MCT1/4 onto the plasma membrane by increased CD147 expression. These findings revealed the pathophysiological roles of activation of P2X7 receptors and enhancement of astrocytic lactate release in ischemic tolerance and could be of keen interest of researchers in the brain ischemia and glia research fields. However, I have several concerns as described below:

1. In Figure 1b, To show that preconditioning (PC)-induced enhanced lactate release does not occur in P2X7 KO mice, a comparison of PC-loaded wild-type and KO mice is not sufficient. The possibility that lactate release in the absence of PC exposure is reduced in P2X7 KO mice compared to wild-type mice and PC-induced enhancement of lactate release itself may be occurring can not be ruled out. The authors should use four groups of mice ([with/without PC exposure] x [wild/KO]).

2. In Figures 2b and 5b, to analyze experimental data with two factors ([control/PC] x [wild/KO] in Fig. 2b and [with/without BzATP] x [control/anti-CD147 IgG] in Fig. 5b), 2-way, not 1-way, ANOVA should be used.

3. Line 219: "ipsilateral striatal section" should be explained in more detail. Did you use whole sections containing the striatum or only the striatum after trimming?

4. Line 317: The term "this increase" makes it difficult to understand whether it is an "increase" in lactate release during severe ischemia compared to the control or an "enhancement of the increase" in lactate release in the group with PC compared to the group without PC.

5. At the beginning of each paragraph in Results section, it is not necessary to explain the results of the previous paragraph (e.g., lines 323-324 and 377-379).

6. Lines 475-476: The sentence "We therefore speculate that the role of CD147 in cerebral ischemia depends on the situation" may be a discussion of the fact that anti-CD147 antibodies exacerbated ischemic injury in the current study, whereas there are reports (Jin et al., 2017; Patrizz et al., 2020) that anti-CD147 antibodies improve ischemic injury. However, "depends on the situation" is not sufficient. The authors should discuss what kind of differences in experimental conditions (timing of administration, dose, and degree of ischemic injury) are related to the different effects of anti-CD147 antibodies.

---------------------------------------------

Rev#2:

The manuscript investigates the mechanism of ischemic tolerance using in vivo and in vitro models. The in vivo experiments were well conducted and are technically not easy. The results of these in vivo experiments, which use microdialysis and perfusion among other methods pinpoint tp aP2X7R mediated mechanism, leading through HIF-1a - CD147 and MCT1,4 to increased lactate release. This release is a major mechanism which helps neurons to survive the insult. The authors demonstrate convincingly that a priming event has to precede this mechanism as it involves not increased glycogen / lactate availability inside astrocytes, but a intracellular shift of the MCT1,4 transporter to the astrocytic cell membrane. The authors expand evidence for their mechanism with the use of primary astrocyte cultures. However, the manuscript is clearly driven by in vivo experiments and the in vitro part is only used to support the proposed mechanism. The manuscript also highlights how reactive astrocytes support challenged neurons. this is an important finding as not everyone agrees that reactive gliosis is beneficial for neuronal survival. Reactive gliosis is clearly a double-edged sword when it comes to neuronal health, but in the case the manuscript highlights a beneficial mechanism with potential therapeutic use of lactate supplements in stroke.

I do not see any serious problems with the approach, the techniques and the analysis. The combination of in vivo with in vitro methods is an asset. The authors are not over-interpretating their results in the discussion, but are careful to point out more experiments are needed for a full proof. Yet, the evidence presented is remarkable and a stepping stone to new insights in preventing ischemic damage.

Minor: on page 7, line 91 the authors refer to lactate as a "transmitter". Neurotransmitters are strictly defined and lactate does not qualify. It should be referred to as signal or modulator. I am not even sure that the word "gliotransmitter" would apply as in all likelihood lactate has a metabolic effect on the energy-starved neurons during an ischemic insult.

## Author Response

Response to Reviewer #1 Thank you for your time and comments regarding our manuscript, and for your detailed suggestions. As described below, we have added additional information to the revised manuscript to address your concerns. Please see our point-by-point responses below for more details.

Comment #1: In Figure 1b, To show that preconditioning (PC)-induced enhanced lactate release does not occur in P2X7 KO mice, a comparison of PC-loaded wild-type and KO mice is not sufficient. The possibility that lactate release in the absence of PC exposure is reduced in P2X7 KO mice compared to wild-type mice and PC-induced enhancement of lactate release itself may be occurring can not be ruled out. The authors should use four groups of mice ([with/without PC exposure] x [wild/KO]).

Response: Thank you for this important comment. To address this concern, we performed an additional in vivo experiment. At present, we have no P2X7-/- mice in our laboratory because of repair work in our animal rooms. We therefore performed an alternative experiment using a centrally permeable P2X7 receptor antagonist, JNJ-47965567. We found that intraperitoneal JNJ-47965567 administration had no effect on extracellular lactate levels with severe MCAO alone (i.e., without PC). We also confirmed that JNJ-47965567 treatment suppressed the PC-induced enhancement of the increase in extracellular lactate levels during severe MCAO. We have added these results as Figure 1-1, and have added the corresponding text to the revised manuscript (Methods, line 167; Results, line 314; References; line 521; and Figure legends: lines 654 and 661).

Comment #2: In Figures 2b and 5b, to analyze experimental data with two factors ([control/PC] x [wild/KO] in Fig. 2b and [with/without BzATP] x [control/anti-CD147 IgG] in Fig. 5b), 2-way, not 1-way, ANOVA should be used.

Response: Thank you for your comment, and we agree with you. In Figures 2b and 5b, we have analyzed the experimental data using two-way ANOVA followed by Tukey's post hoc multiple comparison test; the same results were found. We have made changes to the corresponding sentences in the revised manuscript (Methods, line 297; Figure legends, lines 682 and 722).

Comment #3: Line 219: "ipsilateral striatal section" should be explained in more detail. Did you use whole sections containing the striatum or only the striatum after trimming? Response: For the quantification of glycogen, we used ipsilateral striatal sections after trimming. We have made changes to the corresponding sentence in the revised manuscript (line 217).

Comment #4: Line 317: The term "this increase" makes it difficult to understand whether it is an "increase" in lactate release during severe ischemia compared to the control or an "enhancement of the increase" in lactate release in the group with PC compared to the group without PC.

Response: Thank you for your comment, and we agree with you. We have changed the corresponding sentences in the revised manuscript (Results, line 311; Figure legends, line 649).

Comment #5: At the beginning of each paragraph in Results section, it is not necessary to explain the results of the previous paragraph (e.g., lines 323-324 and 377-379).

Response: Thank you for your comment. We have deleted the corresponding sentences from the revised manuscript (lines 324, 365, and 377).

Comment #6: Lines 475-476: The sentence "We therefore speculate that the role of CD147 in cerebral ischemia depends on the situation" may be a discussion of the fact that anti-CD147 antibodies exacerbated ischemic injury in the current study, whereas there are reports (Jin et al., 2017; Patrizz et al., 2020) that anti-CD147 antibodies improve ischemic injury. However, "depends on the situation" is not sufficient. The authors should discuss what kind of differences in experimental conditions (timing of administration, dose, and degree of ischemic injury) are related to the different effects of anti-CD147 antibodies.

Response: Thank you for your comment; we agree with you. We have made changes to the corresponding sentence in the revised manuscript (line 473).

Response to Reviewer #2 Comment: Minor: on page 7, line 91 the authors refer to lactate as a "transmitter". Neurotransmitters are strictly defined and lactate does not qualify. It should be referred to as signal or modulator. I am not even sure that the word "gliotransmitter" would apply as in all likelihood lactate has a metabolic effect on the energy-starved neurons during an ischemic insult.

Response: Thank you for your time and encouraging comments. We agree with your suggestion and have altered this text in the revised version of the manuscript (line 86).
