# Supplementary figures and images for: Preconditioning-Induced Facilitation of Lactate Release from Astrocytes Is Essential for Brain Ischemic Tolerance
Source: eNeuro. 2024 Apr 23;11(4):ENEURO.0494-23.2024. doi: 10.1523/ENEURO.0494-23.2024 (PMC11064122; doi:10.1523/ENEURO.0494-23.2024)

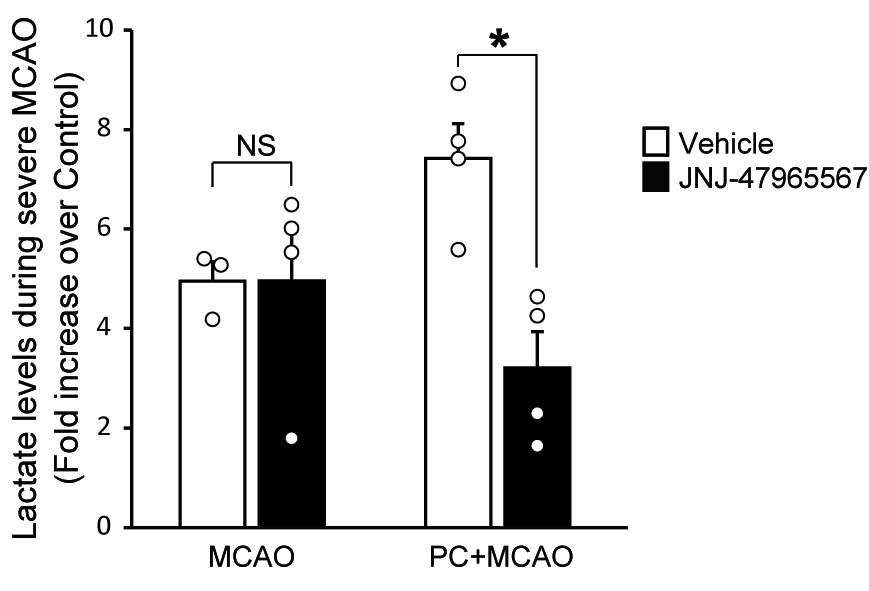

Supplement: Figure 1-1 — The P2X7 receptor antagonist JNJ-47965567 suppressed the preconditioning (PC)-induced enhancement of increased extracellular lactate levels during severe ischemia. JNJ-47965567 (30 mg/kg) was intraperitoneally injected 1 day before severe middle cerebral artery occlusion (MCAO; for 1 h). Although intraperitoneal JNJ-47965567 administration had no effects on extracellular lactate levels during severe MCAO alone (i.e., without PC [15 min of MCAO]), this treatment suppressed the PC-induced enhancement of the increase in extracellular lactate levels during severe MCAO. Data show the fold increase over control (naïve) mice. Values are shown as means ± SEM; *P < 0.05, two-way ANOVA followed by Tukey’s post hoc multiple comparison test; n = 3–4. NS, not significant. Download Figure 1-1, TIF file. [file eneuro-11-ENEURO.0494-23.2024-s001.tif]
